# Interferon Lambda 4 Genotype Is Not Associated with Recurrence of Oral or Genital Herpes

**DOI:** 10.1371/journal.pone.0138827

**Published:** 2015-10-02

**Authors:** Krystle A. Lang Kuhs, Mark H. Kuniholm, Ruth M. Pfeiffer, Sabrina Chen, Seema Desai, Brian R. Edlin, Marion G. Peters, Michael Plankey, Gerald B. Sharp, Howard D. Strickler, Maria C. Villacres, Thomas C. Quinn, Stephen J. Gange, Ludmila Prokunina-Olsson, Ruth M. Greenblatt, Thomas R. O’Brien

**Affiliations:** 1 Infections and Immunoepidemiology Branch, Division of Cancer Epidemiology and Genetics, National Cancer Institute, NIH, Bethesda, Maryland, United States of America; 2 Department of Epidemiology and Population Health, Albert Einstein College of Medicine, Bronx, New York, United States of America; 3 Biostatistics Branch, Division of Cancer Epidemiology and Genetics, National Cancer Institute, NIH, Bethesda, Maryland, United States of America; 4 Information Management Services, Calverton, Maryland, United States of America; 5 Department of Immunology and Microbiology, Rush University Medical Center, Chicago, Illinois, United States of America; 6 Institute for Infectious Disease Research, National Development and Research Institutes, New York, New York, United States of America; 7 Department of Medicine, University of California San Francisco, San Francisco, California, United States of America; 8 Division of Infectious Diseases, Georgetown University Medical Center, Washington, D.C., United States of America; 9 Epidemiology Branch, Division of AIDS, National Institute of Allergy and Infectious Diseases, Bethesda, Maryland, United States of America; 10 Department of Pediatrics, University of Southern California, Los Angeles, California, United States of America; 11 Division of Intramural Research, National Institute of Allergy and Infectious Diseases, NIH, Bethesda, Maryland, United States of America; 12 Department of Epidemiology, Johns Hopkins Bloomberg School of Public Health, Baltimore, Maryland, United States of America; 13 Laboratory of Translational Genomics, Division of Cancer Epidemiology and Genetics, National Cancer Institute, NIH, Bethesda Maryland, United States of America; 14 Departments of Clinical Pharmacy, Medicine, Epidemiology, and Biostatistics, University of California San Francisco, San Francisco, California, United States of America; McMaster University, CANADA

## Abstract

*IFNL4*-ΔG/TT (rs368234815) genotype is associated with hepatitis C virus clearance and may play a role in other infections. IFN-λ4 protein is generated only in individuals who carry the *IFNL4-*ΔG allele. The *IFNL4* rs12979860-T allele, which is in strong linkage disequilibrium with *IFNL4-*ΔG, was recently reported to be associated with more frequent and severe oral herpes episodes. We investigated the association of *IFNL4-*ΔG/TT with herpes simplex virus (HSV)-related outcomes among 2,192 African American and European American participants in the Women’s Interagency HIV Study (WIHS). WIHS is a prospective cohort study of human immunodeficiency virus (HIV)–infected and at-risk women that began in 1994. This report includes follow-up through 2013. Available data included: HSV–1 and HSV–2 antibodies at study entry; bi-annually ascertained episodes of (self-reported) oral herpes, (self-reported) genital sores and (clinician-observed) genital ulcers; HSV–2 DNA in cervicovaginal lavage (CVL) specimens. *IFNL4*-ΔG/TT genotyping was determined by TaqMan. We compared women with *IFNL4*-ΔG/ΔG or *IFNL4*-TT/ΔG genotypes (i.e., *IFNL4-*ΔG carriers) to those with the *IFNL4*-TT/TT genotype, adjusting for age, race and HIV status. For outcomes with repeated measurements, the adjusted odds ratio (aOR), 95% confidence interval [CI] and p-value were determined using a generalized estimating equations approach. Median participant age at enrollment was 36 years; 81% were African American, 74% were HIV-infected. Among 1,431 participants tested for antibodies, 72.8% were positive for HSV–1 and 79.0% were positive for HSV–2. We observed no association between *IFNL4-*ΔG/TT genotype and any outcome: HSV–1 or HSV–2 antibody prevalence (p>0.1, all comparisons); oral herpes (aOR, 1.2; p = 0.35); genital sores (aOR, 1.0; p = 0.71); genital ulcers (aOR, 1.1; p = 0.53); detectable HSV–2 DNA in CVL (N = 322; aOR, 0.71; p = 0.49); HSV–2 DNA level (p = 0.68). In this large prospective study, *IFNL4-*ΔG/TT genotype was not associated with HSV-related outcomes, including episodes of oral or genital herpes.

## Introduction

**Fig 1:**
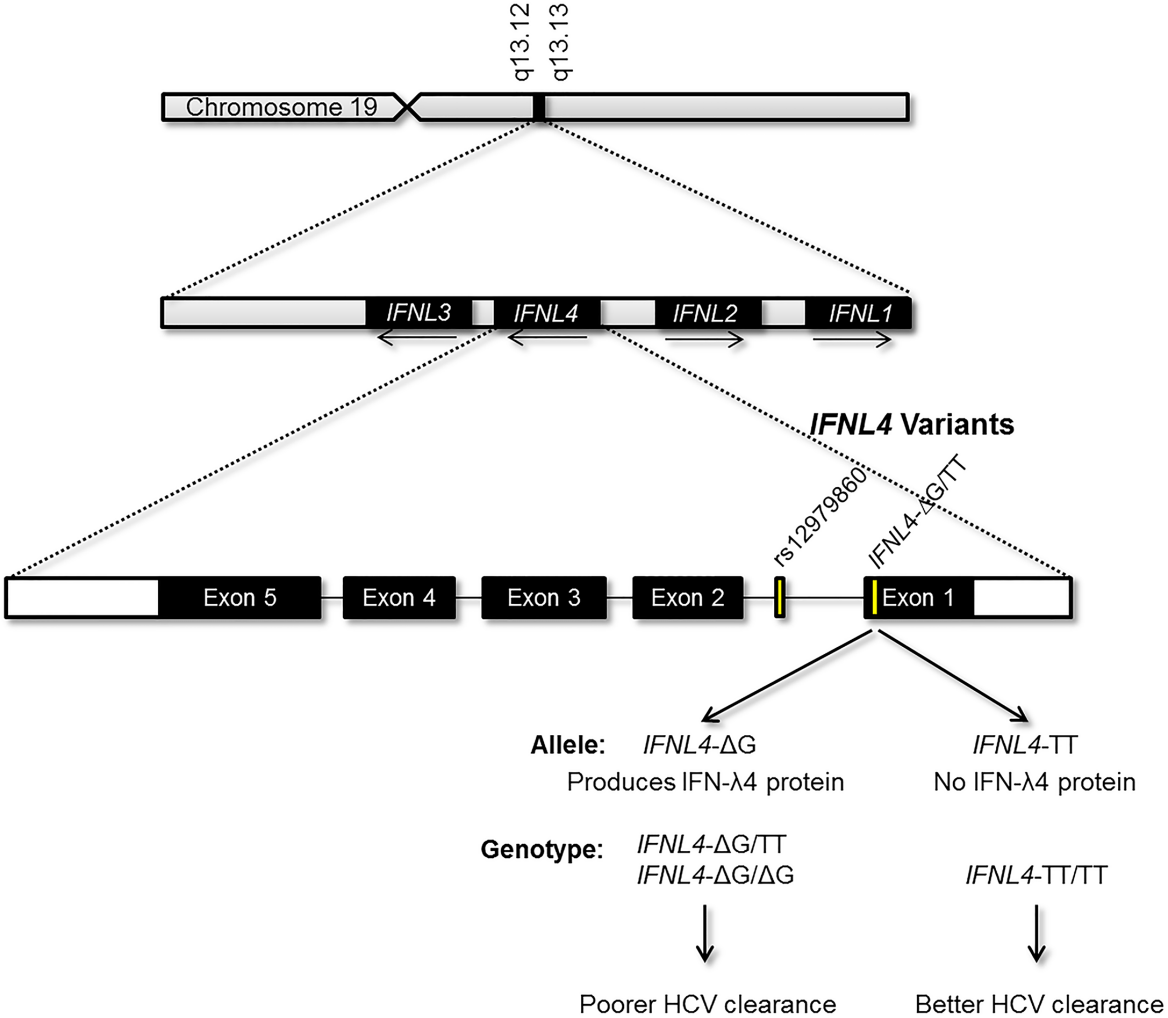
Depiction of *IFNL4* within the interferon lambda region on chromosome 19, as well as the exonic structure of the gene and the location of the rs12979860 and *IFNL4*-∆G/TT genetic variants. Only carriers of the *IFNL4*-ΔG allele, which has been associated with poorer clearance of HCV infection, are able to produce the IFN-λ4 protein.

Genome-wide association studies (GWAS) have identified variants within the interferon lambda (IFN-λ) region that are strongly associated with both spontaneous clearance of hepatitis C virus (HCV) and response to treatment for chronic hepatitis C [1–5]. A recently discovered common variant (rs368234815, previously ss469415590) within the novel interferon lambda 4 gene (symbol: *IFNL4*) appears to account for these associations through control of the IFN-λ4 protein [6] (Fig 1). We here refer to the rs368234815 polymorphism as *IFNL4*-ΔG/TT. The IFN-λ4 protein is only produced by individuals who carry at least one copy of the *IFNL4*-ΔG allele, which is associated with impaired HCV clearance. The alternative *IFNL4*-TT allele creates a frameshift in exon 1; individuals who are homozygous for *IFNL4*-TT do not generate IFN-λ4 and have enhanced HCV clearance. Previously, we demonstrated that *IFNL4-*ΔG/TT genotype is a stronger predictor of HCV clearance than genotype for rs12979860, a single nucleotide polymorphism (SNP) marker used in GWAS. Although it is commonly called an ‘IL28B’ variant, rs12979860 actually lies within the first intron of *IFNL4*. The unfavorable rs12979860-T allele is in strong linkage disequilibrium with the *IFNL4*-ΔG allele; therefore, it is likely that rs12979860-T acts only as a marker for *IFNL4*-ΔG. Thus, *IFNL4*-ΔG/TT is the leading candidate for the primary functional variant underlying the observed genetic associations with HCV clearance [6–8].

There is strong evidence that genotype for *IFNL4-*ΔG/TT may play a role in other infections [9, 10] and, given that expression of the interferon lambda receptor (IFN-λR1) is largely restricted to cells of epithelial origin [11, 12], it is plausible that this genetic variant could affect viral infections of the epithelium. A recent report implicated *IFNL4-*ΔG as a possible risk factor for recurrent herpes episodes [13]. Griffiths et al demonstrated that the anti-viral effects of Med23 on HSV–1 replication were mediated through upregulated mRNA and protein expression of interferon lambda family members. Griffiths et al also reported in a case-control analysis of 56 Italian subjects that the rs12979860-T allele was associated with more frequent and severe oral herpes simplex virus (HSV) episodes. [6]. The goal of our study was to investigate the association of *IFNL4-*ΔG/TT genotype with the frequency of oral and genital herpes episodes within the large prospective Women’s Interagency HIV Study (WIHS).

## Materials and Methods

### Study Population

WIHS is a cohort study of human immunodeficiency virus (HIV)–infected and at-risk HIV-seronegative women who were enrolled at 6 clinical sites [14]. Initial enrollment was conducted during 1994–1995, a second recruitment occurred during 2001–2002, and a third recruitment period started in 2011 (women enrolled in 2011- cohort are not included in this analysis). Every 6 months women were administered a detailed health questionnaire and underwent a clinical examination with collection of blood specimens. This analysis is based on data collected through 2013. This study focused on the 2,192 women who were successfully genotyped for IFNL4-ΔG/TT and who, on the basis of self-reported information on race/ethnicity, were of African American or European American ancestry, but not of Hispanic ethnicity. The WIHS protocol was approved by each local institutional review board (IRB), and all participants included in this analysis provided written informed consent for genetic testing.

### Ethics Statement

The National Institutes of Health (NIH) Office of Human Subjects Research (OHSR) deemed this study excluded from IRB review per 45 CFR 46.102. All human samples and data were analyzed anonymously. All data used in the present analysis are contained within previously published studies [8, 15, 16].

### Ascertainment of Herpes Episodes

Information regarding oral herpes episodes that occurred since the previous study visit was collected by interview in the women who were enrolled in 1994–1995 (information was collected up to and including bi-annual visit 14). Self-reported episodes of genital herpes between study visits were ascertained at all visits for both the 1994–1995 and 2001–2002 enrollees. As part of each 6 month study visit, clinicians assessed the presence of genital herpes based on visualization of ulcers or vesicles during gynecological examination [15].

### Laboratory

#### HSV–1 and HSV–2 Antibodies

Testing for HSV type-specific antibodies was performed on samples collected from women in the 1994–1995 enrollment cohort only [17] using a type 1 and type 2 glycoprotein G-based enzyme immunoassay (Gull Laboratories, Salt Lake City, Utah, USA). Sera with negative or equivocal results were tested by Western blot.

#### HSV–2 DNA Viral Titer Testing

In a subset of HIV-infected women, cervicovaginal lavage (CVL) specimens collected at enrollment were tested for HSV–2 DNA as previously described [16]. CVL specimens were collected by flushing the cervix with 10 mL sterile normal saline; 200μL of each specimen was tested for HSV–2 DNA using duplex quantitative realtime TaqMan polymerase chain reaction (qPCR).

#### *IFNL4*-ΔG/TT and *IFLN4* rs12979860 Genotyping

Genotyping for *IFNL4*-ΔG/TT (rs368234815) and rs12979860 was performed at the Laboratory of Translational Genomics, National Cancer Institute with custom TaqMan allelic discrimination genotyping assays, as previously described [6]. For quality control, blinded duplicate specimens were included in the panel.

### Statistical Analysis

*IFNL4*-ΔG/TT genotype frequency varies markedly by racial ancestry [6]; therefore, all analyses were either stratified by self-reported race/ethnicity or adjusted for that variable. Analyses compared *IFNL4*-ΔG carriers (i.e., women with either the *IFNL4*-ΔG/ΔG or *IFNL4*-ΔG/TT genotype) to those with the *IFNL4*-TT/TT genotype. Seroprevalence of antibodies to HSV–1 and HSV–2 was compared by *IFNL4*-ΔG/TT genotype on the basis of p-values that were calculated by the Mantel-Haenszel chi-square test. The frequency of self-reported and clinically observed herpes episodes were calculated as the proportion of total visits. To calculate unadjusted and adjusted odds ratio (aOR) and 95% confidence intervals (CIs) for the frequency of herpes episodes by *IFNL4*-ΔG/TT genotype, we used logistic regression to separately model each outcome using a binary variable, Y_ij_, which equaled one if the i^th^ woman reported having had the outcome at the j^th^ visit and otherwise equaled zero. To accommodate correlations of observations for the same woman in the variance of the estimates, we used a generalized estimating equation (GEE) approach [18] with an independent working correlation matrix. The adjusted GEE models included age and HIV status as covariates because these variables have been associated with the frequency of herpes recurrence; models that combined both racial ancestry groups also controlled for race. We performed several sensitivity analyses: i) restricting the oral herpes analysis to women who were positive for HSV–1 antibodies; ii) restricting analyses of genital sores and genital ulcers to women who were positive for HSV–2 antibodies; iii) stratifying by HIV status; iv) substituting rs12979860 genotype for *IFNL4*-ΔG/TT genotype in the analysis of oral herpes episodes.

To compare the proportions of women with viral shedding (defined as detectable HSV–2 DNA in the CVL specimen) by *IFNL4*-ΔG/TT genotype, we calculated aORs, 95% CIs and p-values by logistic regression. Adjusted models included HIV status and race as covariates as these variables were associated with viral shedding previously [16]. Among the subset of women shedding virus, p-values for differences in the distribution of HSV–2 DNA levels titers by genotype were determined with the Kruskal-Wallis test.

## Results

### Demographic and Clinical Characteristics of the Study Population

The baseline demographic and clinical characteristics of the participants are presented in Table 1. A total of 2,192 participants were included in this analysis; 1,511 participants were enrolled in the 1994–95 cohort (1,169 [77.4%] African American, 342 [22.6%] European American) and 681 were enrolled in 2001–2002 cohort (598 [87.8%] African American, 83 [12.2%] European American). Overall, the median age at enrollment was 36 years, the majority (74.2%) was HIV positive and the median lifetime number of sex partners was 12 (IQR: 6–50).

**Table 1:** Participant characteristics at enrollment.

	Cohorts Combined	1994–1995 Cohort
	N = 2,192	N = 1,511
*Characteristics*		
**Age** (years)		
Median	36	37
IQR	30–41	31–42
**Race**		
African American	80.6%	77.4%
European American	19.4%	22.6%
**HSV Serostatus**		
HSV–1	—	72.8%
HSV–2	—	79.0%
HSV–1 or HSV–2	—	94.5%
**HIV Status**		
Seropositive	74.2%	79.2%
Seronegative	25.8%	20.8%
**CD4 Nadir** ^1^		
<200	40.4%	46.0%
≥200	59.6%	54.0%
**Lifetime Male Partners**		
Median (IQR)	12	15
IQR	6–50	6–50
**History of Injection Drug Use**		
Yes	32.0%	41.5%
No	68.0%	58.5%

^1^Among HIV-infected individuals

### HSV Seroprevalence by IFNL4-ΔG/TT Genotype

HSV antibody data were available for 1,431 (94.7%) of the 1,511 women enrolled in 1994–1995, representing 65.3% of our full analytic cohort. In this group, 72.8% of women were HSV–1 seropositive (77.3% of African Americans and 57.3% of European Americans) and 79.0% were HSV–2 seropositive (83.9% of African Americans and 62.2% of European Americans). The vast majority of women was seropositive for either HSV–1 or HSV–2 (94.5% overall; 97.4% of African Americans and 84.5% of European Americans); 57.4% were seropositive for both HSV–1 and HSV–2.

The prevalence of antibodies to HSV–1 and HSV–2 did not vary significantly by *IFNL4-ΔG/TT* genotype among either African American or European American participants (Table 2). Among African American women, the proportion positive for either HSV–1 or HSV–2 was 97.0% in those with the *IFNL4*-TT/TT genotype and 97.4% in the *IFNL4*-ΔG allele carriers (P = 0.75). Among European American participants, those proportions were 86.6% and 83.1%, respectively (p = 0.39). Similar results were seen for genotype comparisons for HSV–1 and HSV–2 separately. Thus, the data provide no evidence that *IFNL4*-ΔG/TT genotype affects susceptibility to HSV infections.

**Table 2:** Prevalence of antibodies to HSV–1 and HSV–2 at enrollment, WIHS 1994–95 cohort, by race and *IFNL4* genotype (N = 1,431).

		**African Americans**					
***IFNL4*-ΔG/TT Genotype**	**Total**	**HSV–1 Positive**	**p-value**	**HSV–2 Positive**	**p-value**	**HSV–1 or HSV–2 Positive**	**p-value**
		N (%)		N (%)			
Overall	1108	857 (77.3)		930 (83.9)	-	1079 (97.4)	
TT/TT	168	124 (73.8)		137 (81.5)		163 (97.0)	
ΔG/TT	505	393 (77.8)		418 (82.8)		488 (96.6)	
ΔG/ΔG	435	340 (78.2)		375 (86.2)		428 (98.4)	
ΔG/ΔG+ΔG/TT	940	733 (78.0)	0.23	793 (84.4)	0.36	916 (97.4)	0.75
		**European Americans**					
***IFNL4*-ΔG/TT Genotype**	**Total**	**HSV–1 Positive**	**p-value**	**HSV–2 Positive**	**p-value**	**HSV–1 or HSV–2 Positive**	**p-value**
		N (%)		N (%)		N (%)	
Overall	323	185 (57.3)		201 (62.2)	-	273 (84.5)	
TT/TT	134	73 (54.5)		90 (67.2)		116 (86.6)	
ΔG/TT	151	87 (57.6)		86 (57.0)		121 (80.1)	
ΔG/ΔG	38	25 (65.8)		25 (65.8)		36 (94.7)	
ΔG/ΔG+ΔG/TT	189	112 (59.3)	0.39	111 (58.7)	0.12	157 (83.1)	0.39

### Oral Herpes Episodes

A total of 1,511 women enrolled in the 1994–95 cohort contributed 9,322 visits to this analysis. An episode of oral herpes was reported at 5.3% of these visits, 3.7% of visits among African Americans and 10.7% of visits among European Americans. There was no evidence that *IFNL4*-ΔG/TT was associated with recurrent oral herpes (Table 3). Comparing women with the *IFNL4*-ΔG/ΔG or *IFNL4*-ΔG/TT genotype (i.e., women who can generate IFN-λ4 protein) to those with the *IFNL4*-TT/TT genotype (i.e., women who cannot generate IFN-λ4) yielded an aOR of 1.1 (95% CI: 0.7–1.7; p = 0.58) in African American women and a similar result in the European American women (aOR, 1.2; 95% CI: 0.8–1.9; p = 0.44). When results for African Americans and European American individuals were combined in an analysis that was adjusted for age, HIV status and race, the aOR was 1.2 (95% CI: 0.8–1.6; p = 0.35).

**Table 3:** Self-reported episodes of oral herpes among women enrolled in WIHS (1994–1995 cohort only), by race and *IFNL4* genotype.

	African Americans (N = 1,169)				European Americans (N = 342)			
	*IFNL4* Genotype				*IFNL4* Genotype			
	TT/TT	ΔG/TT	ΔG/ΔG	ΔG/TT + ΔG/ΔG	TT/TT	ΔG/TT	ΔG/ΔG	ΔG/TT + ΔG/ΔG
**Oral Herpes** ^1^								
Episodes (No.)	37	113	120	233	83	97	42	139
Visits (No.)	1094	3285	2872	6157	869	978	224	1202
Episodes/visits	0.034	0.034	0.042	0.038	0.096	0.099	0.188	0.116
Adjusted Odds Ratio (95% CI)^2^	ref			1.1 (0.7–1.7)	ref			1.2 (0.8–1.9)
p-value	-			0.58	-			0.44

^1^Study visits 1–14; bi-annual visits from 1994 to 2013

^2^Adjusted for age and HIV status

Linkage disequilibrium (r^2^) between *IFNL4*-ΔG and rs12979860-T, the variant examined in the previous study of genetic associations with oral herpes [13], was 0.99 in European Americans and 0.87 in African Americans, therefore, we observed similar results (aOR, 1.1; 95% CI: 0.8–1.6) for rs12979860 genotype. Likewise, sensitivity analyses that were stratified by HIV status or restricted to women who were HSV–1 seropositive yielded similarly non-significant results (S1 Table). In contrast to the null genotype findings for the *IFNL4*-ΔG/TT variant, women who were infected with HIV reported oral herpes episodes much more frequently than HIV-uninfected women (aOR, 4.31; 95% CI: 2.84–6.55; p<0.0001), thus verifying the validity of the self-reported data. Our results, therefore, provide no support for the hypothesis that the *IFNL4*-ΔG allele increases the risk of recurrent oral herpes.

### Genital Herpes Episodes

A total of 2,192 women contributed 44,828 visits for the analysis of genital herpes (Table 4). At 7.5% of visits, a woman reported that she had suffered an episode of genital herpes during the previous 6 months. Genital herpes was reported at 8.2% of visits by African American women and 8.7% of visits by European Americans. *IFNL4*-ΔG/TT genotype was not associated with frequency of self-reported genital herpes episodes (Table 4) with an aOR of 1.1 (95% CI: 0.9–1.4; p = 0.55) for African Americans and 1.0 (95% CI: 0.7–1.4; p = 0.99) for European Americans comparing *IFNL4*-ΔG/ΔG or *IFNL4*-ΔG/TT genotype to *IFNL4*-TT/TT genotype; results for African and European Americans combined were: aOR 1.0 (95% CI: 0.9–1.3; p = 0.71).

**Table 4:** Self-reported and clinician-observed episodes of genital herpes among women enrolled in WIHS (both cohorts), by *IFNL4* genotype and race.

	African Americans (N = 1767)				European Americans (N = 425)			
	*IFNL4* Genotype				*IFNL4* Genotype			
	TT/TT	ΔG/TT	ΔG/ΔG	ΔG/TT + ΔG/ΔG	TT/TT	ΔG/TT	ΔG/ΔG	ΔG/TT + ΔG/ΔG
**Genital Sores** (Self Reported)								
Episodes (No.)	354	1205	1105	2310	316	343	59	402
Visits (No.)	4979	16621	15019	31640	3618	3754	837	4591
Episodes/visits	0.071	0.073	0.074	0.073	0.087	0.091	0.071	0.088
Adjusted Odds Ratio (95% CI)^1^	ref			1.1 (0.9–1.4)	ref			1.0 (0.7–1.4)
p-value	-			0.55	-			0.99
**Genital Ulcers** ^2^ (Clinician Observed)								
Episodes (No.)	104	370	370	740	51	47	8	55
Visits (No.)	4699	15743	14283	30026	3314	3436	759	4195
Episodes/visits	0.022	0.024	0.026	0.025	0.015	0.014	0.011	0.013
Adjusted Odds Ratio (95% CI)^1^	ref			1.2 (0.9–1.6)	ref			0.8 (0.5–1.4)
p-value	-			0.31	-			0.50

^1^Adjusted for age and HIV status

^2^Missing participants: African Americans, 6; European American, 1

A genital ulcer was noted during clinical exam at 2.2% of visits, 2.4% and 1.4% of visits for African Americans and European Americans, respectively (Table 4). Similar to the results for self-reported genital herpes, the frequency of clinician-observed genital ulcers did not vary by genotype, with an aOR of 1.2 (95% CI: 0.9–1.6; p = 0.31) for African Americans and 0.8 (95% CI: 0.5–1.4; p = 0.50) for European Americans comparing *IFNL4*-ΔG/ΔG or *IFNL4*-ΔG/TT genotype to *IFNL4*-TT/TT genotype; results for African and European Americans combined were: aOR 1.1 (95% CI: 0.8–1.4; p = 0.53). Similar to the results for oral herpes, we found no evidence that the frequency of genital herpes varies by *IFNL4*-ΔG/TT genotype.

### HSV–2 DNA in CVL Specimens

We also examined the relationship between *IFNL4*-ΔG/TT genotype and results of HSV–2 DNA measurements in CVL samples taken from a subset of 322 women (Table 5). In these participants, 27 (8.4%) individuals had detectable HSV–2 viral DNA (i.e., viral shedding). The proportion of women shedding HSV–2 virus did not differ by *IFNL4*-ΔG/TT genotype (aOR, 0.7; 95% CI: 0.3–1.9; p = 0.49, comparing *IFNL4*-ΔG/ΔG or *IFNL4*-ΔG/TT genotype to *IFNL4*-TT/TT genotype). Among the women with detectable HSV–2 DNA, the median viral level was 4.4 log_10_ DNA copies/mL for *IFNL4*-ΔG carriers and 3.2 log_10_ DNA copies/mL for those with the *IFNL4*-TT/TT genotype (p = 0.6831). These results provide no evidence that *IFNL4*-ΔG/TT genotype affects shedding of HSV–2 DNA.

**Table 5:** Presence of HSV–2 DNA and HSV–2 DNA levels in cervical lavage specimens from HIV-infected and uninfected women enrolled in WIHS, by *IFNL4-*ΔG/TT genotype (N = 322).

	**HSV–2 Viral Shedding**			
***IFNL4-*ΔG/TT Genotype**	*Total*	*Shedding HSV–2*, *N(%)*	*Adjusted Odds Ratio* ^1^ *(95% CI)*	*p-value*
TT/TT	61	6 (9.8)	Ref	-
ΔG/TT	161	15 (9.3)		
ΔG/ΔG	100	6 (6.0)		
ΔG/ΔG+ΔG/TT	261	21 (8.0)	0.7 (0.3–1.9)	0.49
	**HSV–2 DNA Level** ^2^			
***IFNL4-*ΔG/TT Genotype**	*Total*	*Median HSV–2 Level (log*_*10*_ *DNA copies/mL)*		*p-value* ^3^
TT/TT	6	3.2		-
ΔG/TT	15	4.4		
ΔG/ΔG	6	3.3		
ΔG/ΔG+ΔG/TT	21	4.4		0.6831

^1^Adjusted for: HIV status and race

^2^Analysis of HSV–2 DNA levels are limited to women with detectable virus

^3^Kruskal-Wallis test (*IFNL4-*TT/TT was used as the reference group)

## Discussion

In this study of more than 2,000 women who were followed prospectively in the WIHS cohort, no associations were observed between *IFNL4*-ΔG/TT genotype and the frequency of self-reported oral herpes, self-reported genital herpes or clinician-observed genital ulcers. Additionally, no associations were observed between *IFNL4*-ΔG/TT genotype and HSV seroprevalence (either HSV–1 or HSV–2); HSV–2 viral shedding or HSV–2 DNA levels in cervical lavage specimens. On the basis of these results, it does not appear that the IFN-λ4 protein plays a role in susceptibility to herpes virus infections or the frequency of recurrence.

We believe ours is the first study to examine the potential association between genetic variants in the IFN-λ region and genital herpes, however, a previous study found rs12979860-T, which is in strong linkage disequilibrium with the *IFNL4*-ΔG allele, to be associated with more frequent and severe episodes of oral herpes [13]. Some differences in the two studies should be noted. Our analysis was based on prospective observation of 1511 women with 15,592 person-visits and, therefore, had considerably greater statistical power than the previous study of 56 subjects. WIHS investigators asked subjects whether they had experienced an episode of oral herpes in the past six months, whereas Griffiths et al [13] categorized patients based on parameters that included the number of episodes/year, the size of the lesion and whether a patient had long lasting symptoms that required antiviral therapy. Therefore, while our large study provides strong evidence that IFN-λ region genetic variants are not associated with the frequency of recurrent oral herpes, it is possible that *IFNL4*-ΔG/TT genotype could play a role in controlling the severity of HSV infection. Given that that *IFNL4*-ΔG is a common allele, this genetic variant might require the presence of other, less common, factors to promote severe recurrent HSV infection. Thus, further investigation of the potential relationship between *IFNL4*-ΔG/TT genotype and severe oral herpes is warranted.

There were several strengths of this study. Our study included more than 2,000 participants with up to 18 years of follow-up time; therefore we were well powered to observe an association between herpes recurrence and *IFNL4*-ΔG/TT genotype if one existed. Additionally, our study was nested within an established cohort with standardized sample collection and procedures as well as reliable clinical ascertainment of genital herpes.

This study provides no evidence that the *IFNL4*-ΔG allele plays a role in oral or genital herpes; however, this variant could influence the pathogenesis of viral infections beyond HCV. The *IFNL4-*ΔG allele, which generates IFN-λ4 protein and is unfavorable for HCV clearance, underwent very strong negative genetic selection with replacement of the ancestral *IFNL4*–ΔG allele by the derived *IFNL4*-TT variant in non-African populations [10]. It seems unlikely that this selection was driven by HCV because the risk factors accounting for the bulk of HCV transmission (blood transfusions, contaminated medical injections and injection drug use) arose primarily in the 20^th^ century and because HCV usually causes a slowly progressive chronic infection that is unlikely to markedly impair host reproduction. It seems likely, therefore, that this selection was driven by one or more other infectious agents. Future studies should focus on the potential role of *IFNL4*-ΔG/TT genotype in other infections.

## Supporting Information

S1 Table Sensitivity analyses for episodes of self-reported oral herpes, self-reported genital sores and clinician-observed genital ulcers among women enrolled in WIHS, by *IFNL4*-ΔG/TT genotype. (DOC)
