# Supplementary material for: Interferon Lambda 4 Genotype Is Not Associated with Recurrence of Oral or Genital Herpes
Source: PLoS One. 2015 Oct 2;10(10):e0138827. doi: 10.1371/journal.pone.0138827 (PMC4592222; doi:10.1371/journal.pone.0138827)
Supplement: S1 Table — (DOC) [file pone.0138827.s001.doc]

| **S1 Table**: Sensitivity analyses for episodes of self-reported oral herpes, self-reported genital sores and clinician-observed genital ulcers among women enrolled in WIHS, by *IFNL4*-ΔG/TT genotype. | | | | | |
| --- | --- | --- | --- | --- | --- |
|  |  |  |  |  |  |
| **Outcome** | **Analysis** | **Total Episodes (No.)** | **Total Visits (No.)** | **Adjusted Odds Ratio**^3^ **(95% CI)** | **p-value** |
| **Oral Herpes**^1^ | HIV-infected | 464 | 7388 | 1.2 (0.9-1.7)^4^ | 0.28 |
|  | HIV-uninfected | 28 | 1934 | 0.7 (0.3-1.7)^4^ | 0.47 |
|  | HSV-1 antibody positive^2^ | 358 | 6494 | 1.2 (0.8-1.7)^5^ | 0.32 |
| **Genital Sores** | HIV-infected | 2994 | 32700 | 1.0 (0.8-1.3)^4^ | 0.77 |
|  | HIV-uninfected | 388 | 12128 | 1.1 (0.7-1.7)^4^ | 0.62 |
|  | HSV-2 antibody positive^2^ | 2386 | 24286 | 1.2 (0.9-1.5)^5^ | 0.14 |
| **Genital Ulcers** | HIV-infected | 864 | 30772 | 1.1 (0.8-1.4)^4^ | 0.69 |
|  | HIV-uninfected | 86 | 11462 | 1.8 (0.8-3.9)^4^ | 0.12 |
|  | HSV-2 antibody positive^2^ | 695 | 22923 | 1.1 (0.8-1.5)^5^ | 0.40 |
|  |  |  |  |  |  |
| ^1^1994-1995 cohort, study visits 1-14 (bi-annual visits from 1994 to 2013)  ^2^1994-1995 cohort only  ^3^All reported odds ratios are for the comparison of *IFNL4*-ΔG/TT + ΔG/ΔG to *IFNL4*-TT/TT  ^4^Adjusted for age and race  ^5^Adjusted for age, HIV status and race | | | | | |
